# Exogenous Hydrogen Peroxide Contributes to Heme Oxygenase-1 Delaying Programmed Cell Death in Isolated Aleurone Layers of Rice Subjected to Drought Stress in a cGMP-Dependent Manner

**DOI:** 10.3389/fpls.2018.00084

**Published:** 2018-02-01

**Authors:** Guanghui Wang, Yu Xiao, Xiaojiang Deng, Heting Zhang, Tingge Li, Huiping Chen

**Affiliations:** ^1^Hainan Key Laboratory for Sustainable Utilization of Tropical Bioresource, Hainan University, Haikou, China; ^2^Institute of Tropical Agriculture and Forestry, Hainan University, Haikou, China

**Keywords:** hydrogen peroxide (H_2_O_2_), heme oxygenase-1 (HO-1), aleurone layer, cyclic guanosine monophosphate (cGMP), drought stress, *Oryza sativa*, programmed cell death (PCD)

## Abstract

Hydrogen peroxide (H_2_O_2_) is a reactive oxygen species (ROS) that plays a dual role in plant cells. Here, we discovered that drought (20% polyethylene glycol-6000, PEG)-triggered decreases of *HO-1* transcript expression and HO activity. However, exogenous H_2_O_2_ contributed toward the increase in *HO-1* gene expression and activity of the enzyme under drought stress. Meanwhile, the HO-1 inducer hematin could mimic the effects of the H_2_O_2_ scavengers ascorbic acid (AsA) and dimethylthiourea (DMTU) and the H_2_O_2_ synthesis inhibitor diphenyleneiodonium (DPI) for scavenging or diminishing drought-induced endogenous H_2_O_2_. Conversely, the zinc protoporphyrin IX (ZnPPIX), an HO-1-specific inhibitor, reversed the effects of hematin. We further analyzed the endogenous H_2_O_2_ levels and *HO-1* transcript expression levels of aleurone layers treated with AsA, DMTU, and DPI in the presence of exogenous H_2_O_2_ under drought stress, respectively. The results showed that in aleurone layers subjected to drought stress, when the endogenous H_2_O_2_ level was inhibited, the effect of exogenous H_2_O_2_ on the induction of HO-1 was enhanced. Furthermore, exogenous H_2_O_2_-activated HO-1 effectively enhanced amylase activity. Application of 8-bromoguanosine 3′,5′-cyclic guanosine monophosphate (8-Br-cGMP) (the membrane permeable cGMP analog) promoted the effect of exogenous H_2_O_2_-delayed PCD of aleurone layers in response to drought stress. More importantly, HO-1 delayed the programmed cell death (PCD) of aleurone layers by cooperating with nitric oxide (NO), and the delayed effect of NO on PCD was achieved via mediation by cGMP under drought stress. In short, in rice aleurone layers, exogenous H_2_O_2_ (as a signaling molecule) triggered HO-1 and delayed PCD via cGMP which possibly induced amylase activity under drought stress. In contrast, as a toxic by-product of cellular metabolism, the drought-generated H_2_O_2_ promoted cell death.

## Introduction

The outer layer of the endosperm is referred to as the aleurone layer in cereal seeds because it contains an abundance of aleurone grains that accumulate proteins. During the process of germination in cereal seeds, the embryo-secreted gibberellic acid (GA) firstly induces the aleurone cells to secrete amylase, protease, and nuclease (Jones and Jacobsen, 1991; Fath et al., 1999), which then degrade the storage compounds of the starchy endosperm into small molecules for embryo growth; finally, the aleurone cells undergo programmed cell death (PCD) after they finish their secretory function. Apparently, the PCD of aleurone layers is promoted by gibberellic acid (GA), while prevented by abscisic acid (ABA) (Bethke et al., 1999; Fath et al., 2000; Xie et al., 2014). Evidence further suggested that H_2_O_2_, heme oxygenase-1 (HO-1, EC.1.14.99.3), and nitric oxide (NO) are involved in GA-induced PCD of aleurone layers (Bethke and Jones, 2001; Beligni et al., 2002; Wu et al., 2016).

H_2_O_2_ is a metabolic product resulting from normal physiological cellular processes. It was previously considered to be toxic in plant cells, and excessive H_2_O_2_ damages biological macromolecules, such as proteins, lipids, and nucleic acids (Larkindale and Huang, 2004). H_2_O_2_ induces cell senescence in the leaves of rice (Hung and Kao, 2004), and participates in GA-induced PCD of the barley aleurone layer and the expression of related resistance genes (Fath et al., 2001). Under non-oxidative stress, exogenous H_2_O_2_ raises the H_2_O_2_ content of the mitochondria in tobacco and *Arabidopsis* cells, resulting in PCD (Maxwell et al., 2002; Tiwari et al., 2002). Fortunately, plants have evolved two types of systems for scavenging H_2_O_2_ to protect against oxidative damage—the enzymatic scavenging system and the non-enzymatic scavenging system. The enzymatic scavenging system includes superoxide dismutase (SOD), catalase (CAT), peroxidase (POD), and ascorbate peroxidase (APX), while the non-enzymatic scavenging system includes ascorbic acid (AsA), glutathione (GSH), and N, N′-dimethylthiourea (DMTU) (Miller et al., 2010; Wu et al., 2011; Kaur et al., 2016). In addition, diphenyleneiodonium (DPI) inhibits NADPH oxidase-mediated production of H_2_O_2_ (Cross and Jones, 1986; Doussière and Vignais, 1992). Increasing numbers of studies have indicated that the appropriate concentration of H_2_O_2_ improves antioxidant enzyme activity (Azevedo-Neto et al., 2005; Li et al., 2011; Hasanuzzaman et al., 2017) and enhances salt resistance (Wahid et al., 2007), drought resistance (Ashraf et al., 2014), and cold resistance (Yu et al., 2002, 2003; Iseri et al., 2013) in plants. Therefore, a fine tuned balance between the production and removal of H_2_O_2_ determines the fate of plant cells.

HO-1 catalyzes the conversion of heme to produce carbon monoxide (CO), biliverdin (BV), and free iron. Biliverdin is subsequently converted to the potent antioxidant bilirubin by biliverdin reductase (Motterlini et al., 2002). In plants, HO-1 plays a physiological role via its catalytic products. For example, BV and CO can protect plants against oxidative stress (Shekhawat and Verma, 2010; Dixit et al., 2014; Fang et al., 2014), and the combined action of HO-1 and BV alleviates the oxidative damage caused by Cd^2+^ stress in soybean leaves (Noriega et al., 2004). HO-1 is induced by heme or its derivatives (hematin and hemin), as well as various stimuli, including ultraviolet-B, H_2_O_2_, and drought (Noriega et al., 2004; Yannarelli et al., 2006; Wu et al., 2016). Our previous study also demonstrated that HO-1 interacts with NO to regulate the PCD of rice aleurone layers subjected to drought stress (Wu et al., 2016). Furthermore, HO-1 delayed the PCD of wheat aleurone layers in collaboration with H_2_O_2_ (Wu et al., 2011). It is well known that HO and HO/CO system play important roles in maintaining the homeostasis of reactive oxygen species (ROS) and protecting cellular function against oxidative damage (Wu and Wang, 2005; Xie et al., 2008; Liu Y.H. et al., 2010). Xuan et al. (2007) reported that CO exerts its physiological function via the 3,5′-cyclic guanosine monophosphate (cGMP) pathway.

It has been demonstrated that exogenous H_2_O_2_ activates both non-enzymatic and enzymatic H_2_O_2_ scavengers in plants under stress conditions (Liu Z.J. et al., 2010; Sathiyaraj et al., 2014; Wang et al., 2014), and HO-1 protects plant cells against oxidative damage (Xie et al., 2008; Wu et al., 2011, 2016). However, whether the levels of endogenous H_2_O_2_ of rice aleurone layers in response to drought stress are lowered by the addition of H_2_O_2_ remains to be identified, and the inter-relationship between HO-1 and H_2_O_2_ in regulating drought-induced PCD of rice aleurone layers is poorly understood. Moreover, the relationships of HO-1, NO, cGMP, and amylase involved in drought stress-induced PCD of rice aleurone layers also requires further investigation.

## Materials and Methods

### Chemicals

All chemicals were obtained from Sigma (St. Louis, MO, United States) unless stated otherwise. In the present study, 20% polyethylene glycol-6000 (PEG) was applied to mimic drought stress. Hematin (an HO-1 inducer) and zinc protoporphyrin IX (ZnPPIX; a specific inhibitor of HO-1) were used at a concentration of 1 and 10 μM, and were dissolved in 0.1 mM NaOH. Sodium nitroprusside (SNP) was used as the NO donor at 200 μM, and 2-(4-carboxyphenyl)-4,4,5,5-tetramethylimidazo-line-1-oxyl-3-oxide (cPTIO) was used as the NO scavenger at 200 μM. Exogenous hydrogen peroxide H_2_O_2_ was used at 1 mM, and diphenyliodonium (DPI), a synthesis inhibitor of H_2_O_2_, was used at 10 μM. Furthermore, N, N′-dimethylthiourea (DMTU, Fluka) and ascorbic acid (AsA) were used as H_2_O_2_ scavengers at a concentration of 5 mM. 8-bromoguanosine 3′,5′-cyclic guanosine monophosphate (8-Br-cGMP) and 1H-[1,2,4]oxadiazolo[4,3-a]quinoxalin-1-one (ODQ) at a concentration of 10 μM were respectively used as a cell-permeable cGMP derivative and the GC inhibitor. The compound 2′,7′-dichlorofluorescin diacetate (H2DCF) was purchased from Calbiochem (La Jolla, CA, United States) and used as an H_2_O_2_-specific fluorescent probe at 50 mM (dissolved in 20 mM CaCl_2_).

### Preparation of Rice Aleurone Layers

Aleurone layers were prepared from de-embryonated rice (*Oryza sativa* L.) grains as described previously (Wu et al., 2016). Firstly, the embryo and distal end were removed from the rice grains, and the embryoless half-grains were sterilized in 0.1% potassium solution for 10 min, and then rinsed with sterile water several times. Finally, the embryoless half-grains were imbibed in Petri dishes with double filter paper containing distilled water, and the dishes were placed into a 25°C constant temperature incubator for 48 h. Aleurone layers were isolated from the cultured grains by removing the starch endosperm. Thereafter, the isolated layers were incubated in 20% PEG alone, or in the absence or presence of 1 mM H_2_O_2_, 1 μM hematin, 10 μM ZnPPIX, 200 μM SNP, 200 μM cPTIO, 10 μM DPI, 5 mM DMTU, 5 mM AsA, 10 μM 8-Br-cGMP, and 2 μM ODQ, and the layers incubated in distilled water were regarded as the control (Con). Based on the experimental requirements, the layers were respectively incubated for 6, 12, 24, and 48 h. All tests were repeated at least three times in independent experiments, with similar results obtained, and 15 or 30 aleurone layers were selected in each replicate.

### Determination of H_2_O_2_ Concentration

The H_2_O_2_ concentration of the crude extract from 15 aleurone layer pieces was determined according to Bellincampi et al. (2000) in the extracellular phase. To determine the H_2_O_2_ concentration, the weighed fresh aleurone layers samples (15 pieces) were firstly extracted with 4 ml clod acetone and centrifuged at 10,000 × *g* for 15 min at 4°C. Then 500 μL supernatant extracted by acetone was mixed with 500 μL reaction liquid containing 500 μM ferrous ammonium sulfate, 50 mM H_2_SO_4_, 200 μM xylenol orange, and 200 μM sorbitol. Following incubation at 30°C for 45 min, the absorbance was measured at 560 nm. The H_2_O_2_ concentration was determined from a calibration curve obtained by adding variable amounts of H_2_O_2_. Data were expressed as μmol H_2_O_2_ per gram of fresh weight of aleurone layers.

### Determination of HO Activity

The HO activity was measured using the method described in our previous report (Wu et al., 2016). Fifteen layers were homogenized with 4 mL of 25 mM HEPES-Tris buffer (pH 7.4) containing 250 mM mannitol, 1 mM EDTA, 1 mM DTT, 1% PVP, and 10% glycerol. The homogenate was centrifuged at 4°C and 2,600 × *g* for 30 min, and then the supernatant was centrifuged at 4°C and 60,000 × *g* for 30 min. The supernatant was used for the determination of HO-1 activity. The reaction mixture (10 mL) contained 40 μL enzyme liquid, 100 mM HEPES-NaOH buffer (pH 7.2), 0.15 mg⋅L^-1^ bovine serum albumin (BSA), 10 μM Hemin, 50 μg⋅L^-1^ ferredoxin, 0.025 mM ferredoxin-NADP^+^ reductase, 5 mM ascorbic acid and 2 mM desferrioxamine, and 100 μM NADPH. The reaction time started from when the NADPH was added, after which the solution was incubated in a water bath at 37°C for 30 min and then placed on ice to terminate the reaction. The increase in BV concentration was determined by the extinction coefficient 6.25 mM⋅cm^-1^ at 650 nm. One unit of activity (U) was defined as the enzyme amount catalyzing the formation of 1 nmol BV per 30 min. Protein content was determined by the Coomassie brilliant blue method (Bradford, 1976) using BSA as a reference standard curve.

### Detection of Amylase Activity

Amylase activity was measured using the 3,5-dinitro salicylic acid procedure (Miller, 1959). Fifteen layers were homogenized with 7 mL distilled water for 20 min, and the homogenate was centrifuged at 5,000 × *g* for 10 min and the supernatant was topped with 50 mL distilled water as the amylase solution. One mL amylase solution was kept in a water bath at a constant 40°C for 10 min, after which 1 mL 10 g⋅L^-1^ starch solution and 2 mL DNS solution were added. After incubation in boiling water for 5 min, distilled water was added to 20 mL and the absorbance was measured at 540 nm. The amylase activity was expressed as the quantity of maltose produced by enzyme catalysis in 1 g of aleurone layer in a minute, and the quantity of maltose production was determined from a calibration curve of maltose in the range of 0.2–2.0 mg.

### Cell Viability Assay

Viability of cells in intact aleurone layers was analyzed according to the method of Wu et al. (2016). The layers were stained with the double fluorescence probe fluorescein diacetate (FDA, 2 μg⋅mL^-1^ in 20 mM CaCl_2_) for 30 min followed by *N*-(3-triethylammoniumpropyl)-4-(6-(4-(diethylamino) phenyl)-hexatrienyl) pyridinium dibromide (FM4-64, 1 μg⋅mL^-1^ in 20 mM CaCl_2_) for 15 min. The stained layers were observed and the images were captured using a laser scanning confocal microscope (LSCM; Olympus, Fluoview 1000). The FV10-ASW 1.6 Viewer software was used with the following parameters: green excitation wavelength at 488 nm; orange excitation wavelength at 586 nm; power 5%; and medium scan. The number of live and dead cells in at least four different fields in a sample was counted to determine the proportion of viable cells.

### Determination of H_2_O_2_ Fluorescence Intensity

The aleurone layers from different treatments were incubated in 50 mM 2′,7′-dichlorofluorescin diacetate (H2DCF; Calbiochem, La Jolla, CA, United States) dissolved in 20 mM CaCl_2_ in a dark room for 25 min, and were then washed to reduce background fluorescence with 20 mM CaCl_2_ twice for 10 min. The images were obtained by a LSCM (Olympus, Fluoview 1000) at an excitation of 488 nm and an emission of 515–530 nm. Relative amount of H_2_O_2_ in the aleurone cells was quantified with the Leica software package.

### Determination of Quantitative Real-Time Fluorescence PCR (qRT-PCR)

qRT-PCR was carried out according to the methods described in our previous work (Wu et al., 2016). Total RNA was isolated from 30 aleurone layer pieces using TRIzol Reagent (Invitrogen, Carlsbad, CA, United States) according to the manufacturer’s instructions. The concentration of RNA was quantified using a UV-1800 spectrophotometer (Shimadzu, Japan). Reverse transcription was performed using the PrimeScript RT reagent Kit (TaKaRa, Dalian, China), following the manufacturer’s procedures. For the first-strand cDNA synthesis, a 20-μL reaction volume was used containing 0.5 μg DNA-free total RNA together with 4 μL 5× PrimeScript Buffer 2, 1 μL RT Primer Mix, and 1 μL PrimeScript RT Enzyme Mix. qRT-PCR was performed using the SYBR Green real-time PCR Master Mix (TianGen, Beijing, China), following the manufacturer’s instructions. The PCR Master Mix per reaction contained 9 μL of 20 × SYBR Green (containing 2.5 × Real Master Mix), 4 μL of cDNA, and 0.5 μL of each oligonucleotide primer. The PCR amplification was performed using the following primers: for EF-1α (GenBank Accession Number: AA753281), forward 5′-ACGGCAAAACGACCAAGAAG-3′ and reverse 5′-CAAGAACGGTGATGTGGTATGG-3′, amplifying a 134-bp fragment; for HO-1 (GenBank Accession Number: CA753857), forward 5′-TCAAGGAACAGGGTCACACAA-3′ and reverse 5′-CCTCCAGCCGTATGAGCAA-3′, amplifying a 142-bp fragment. The relative expression levels of HO-1 were presented as values relative to the corresponding control samples at 0 h, after normalizing against the transcript levels of EF-1α.

### Statistical Analysis

The results reflect the means ±*SE* of at least three independent experiments, and Duncan’s multiple test (*P* < 0.05) was used to consider statistical significant.

## Results

### Exogenous H_2_O_2_ Promoted *HO-1* Transcription and HO Activity in Rice Aleurone Layers Subjected to Drought Stress

**FIGURE 1:**
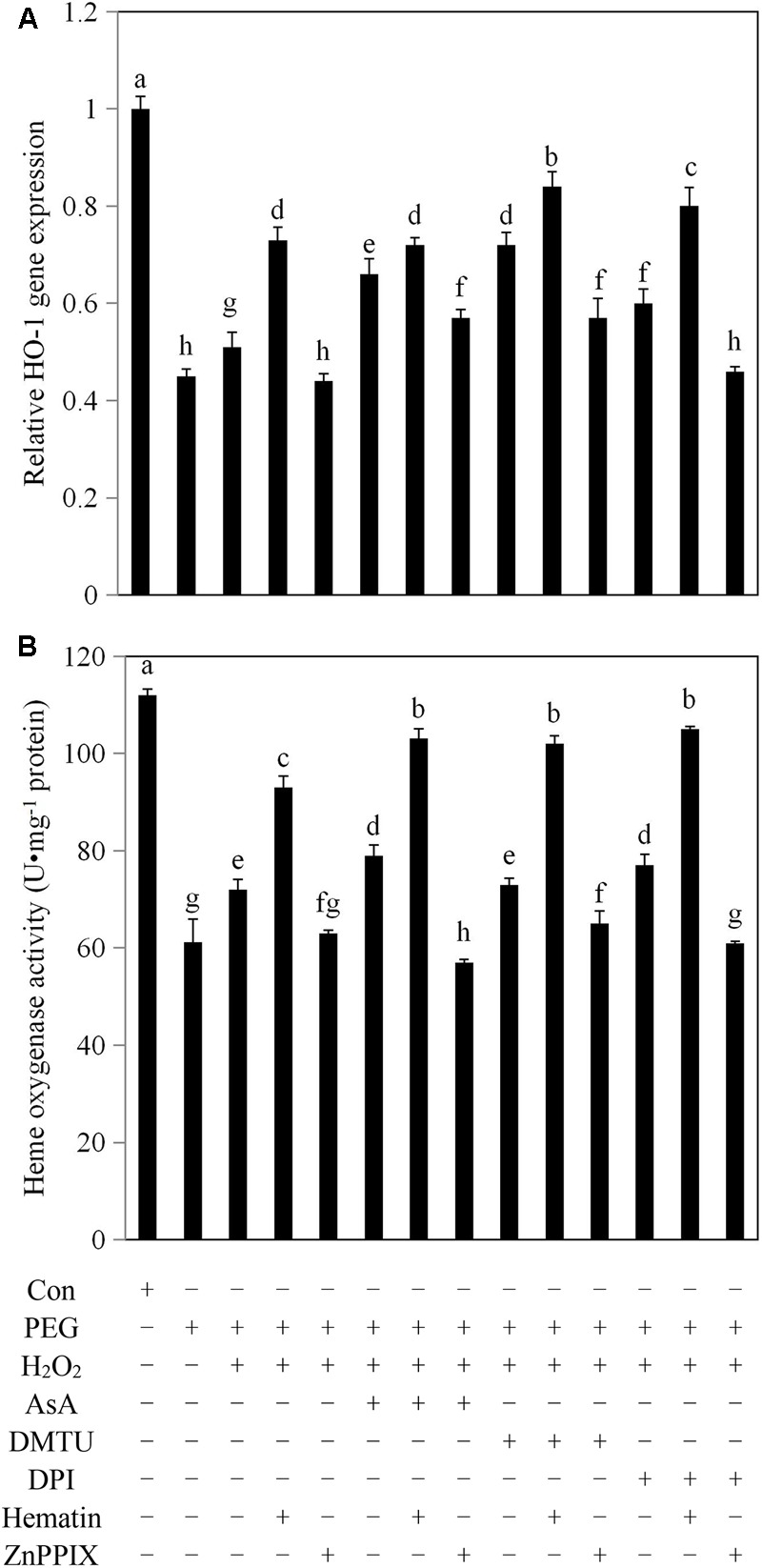
The addition of exogenous H_2_O_2_ enhanced *HO-1* transcript levels and HO activity in rice aleurone layers under drought stress. Rice aleurone layers were incubated in a solution containing distilled water (Con) alone or 20% PEG, 1 mM H_2_O_2_, 5 mM AsA, 5 mM DMTU, 10 μM DPI, 1 μM hematin, and 10 μM ZnPPIX alone or a combination thereof for 6 h. *HO-1* gene expression transcript **(A)** and HO activity **(B)** were detected after 6 h of various treatments, respectively. Data represent the means ± SD of three independent experiments with at least three replicates for each. Within each set of experiments, bars denoted by the different letters are significantly different to the Con treatment at *P* < 0.05 according to Duncan’s multiple test.

HO-1 is an inducible HO that plays an important physiological role in oxidation protection, and also constitutes a type of antioxidant enzyme. As shown in **Figure 1A**, *HO-1* transcript expression in the aleurone cells treated with PEG alone decreased by 55% compared with aleurone cells undergoing a distilled water treatment for 6 h, indicating that drought significantly inhibited the transcript expression of *HO-1* in rice aleurone layers. However, in the treatment of PEG plus exogenous H_2_O_2_, the expression of the *HO-1* transcript increased by 13% compared with PEG treatment alone. The result implies that the *HO-1* transcript expression of rice aleurone layers is induced by exogenous H_2_O_2_ during drought stress. The transcript levels of *HO-1* were increased by 43, 29, 41, and 18% after PEG + H_2_O_2_ + hematin, PEG + H_2_O_2_ + AsA, PEG + H_2_O_2_ + DMTU, and PEG + H_2_O_2_ + DPI treatments, respectively, compared with the PEG + H_2_O_2_ treatment (**Figure 1A**). The result indicates that the HO-1 inducer hematin, the H_2_O_2_ scavengers AsA and DMTU, and the H_2_O_2_ synthesis inhibitor DPI significantly promote the expression of the *HO-1* gene in rice aleurone layers subjected to drought stress. Furthermore, the HO-1 inducer hematin had a superposition effect on the *HO-1* transcript level in combination with AsA, DMTU, and DPI, and the obtained evidence confirmed that the levels of *HO-1* expression were increased in the aleurone layers treated with PEG + H_2_O_2_ + AsA + hematin, PEG + H_2_O_2_ + DMTU + hematin, and PEG + H_2_O_2_ + DPI + hematin. The effects were reversed by the corresponding treatments when the HO-1-specific inhibitor ZnPPIX was added (**Figure 1A**).

The HO activity in aleurone layers treated with PEG alone decreased 45% compared with the layers treated with distilled water, and the HO activity of the layers treated with PEG + H_2_O_2_ increased by 18% compared with the layers treated with PEG alone (**Figure 1B**), suggesting that the HO activities of the layers were significantly inhibited by drought stress and that the exogenous H_2_O_2_ contributed toward the increase in the HO activity in the drought stress-induced aleurone layers. When the H_2_O_2_ scavengers AsA and DMTU and the H_2_O_2_ synthesis inhibitor DPI were added to the treatment with PEG + H_2_O_2_, DMTU exhibited little effect on the activity of HO, and AsA and DPI increased HO activities to different degrees (10 and 7%, respectively). Conversely, when hematin was added to the PEG + H_2_O_2_, PEG + H_2_O_2_ + AsA, PEG + H_2_O_2_ + DMTU, and PEG + H_2_O_2_ + DPI treatments, the HO activities were significantly higher than those treatments without hematin, but the increased activities were suppressed following the addition of ZnPPIX (**Figure 1B**).

### Exogenous H_2_O_2_ Reduced the Amount of Endogenous H_2_O_2_ in the Aleurone Layers Subjected to Drought Stress

**FIGURE 2:**
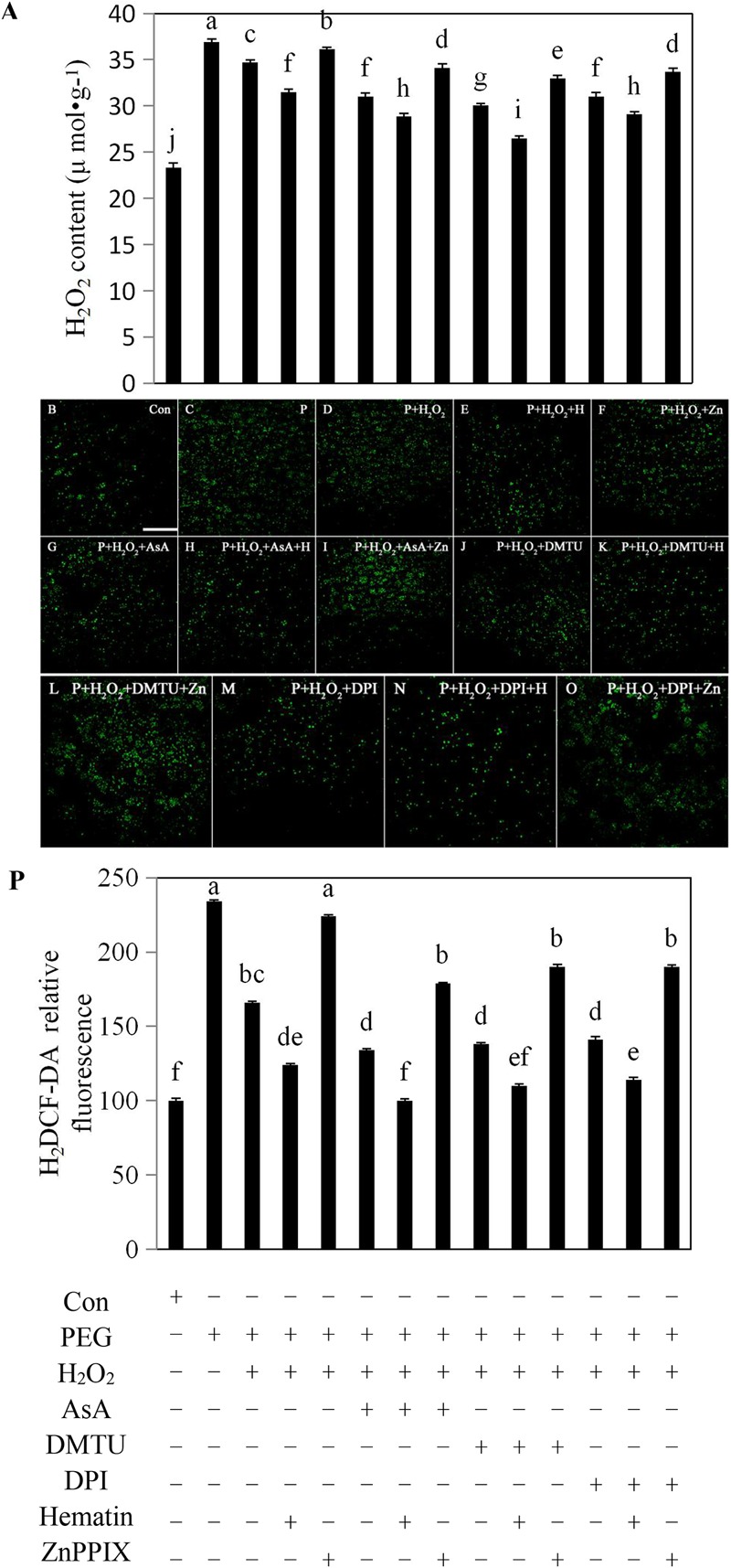
Exogenous H_2_O_2_, hematin, AsA, DMTU, and DPI reduced the production of endogenous H_2_O_2_ in rice aleurone layers under drought stress. Rice aleurone layers were incubated in a solution containing distilled water (Con) alone or 20% PEG, 1 mM H_2_O_2_, 5 mM AsA, 5 mM DMTU, 10 μM DPI, 1 μM hematin, and 10 μM ZnPPIX alone or a combination thereof for 24 h. After various treatments, the contents of H_2_O_2_ **(A)** were detected, meanwhile, the aleurone layers stained with H_2_DCF-DA were observed by LSCM, and then images of the distribution of H_2_O_2_ in fluorescently labeled aleurone cells were immediately captured **(B–O)**. The relative intensity of H_2_DCF-DA fluorescence in the corresponding aleurone layers was also established **(P)**. Scale bar, 100 μM. Data are the means ± SD of three independent experiments with at least three replicates for each. Within each set of experiments, bars denoted by the different letters are significantly different to the Con treatment at *P* < 0.05 according to Duncan’s multiple test.

To explore whether H_2_O_2_ plays a critical role in regulating the amount of endogenous H_2_O_2_, we assessed H_2_O_2_ contents in the aleurone layers under drought stress. Treatment with PEG increased the cellular H_2_O_2_ content of the layer by 59% compared with the distilled water culture, and the addition of exogenous H_2_O_2_ to the PEG treatment decreased the cellular H_2_O_2_ content (**Figure 2A**). Therefore it is possible that exogenous H_2_O_2_ acts as a signaling molecule by triggering the H_2_O_2_ scavenging mechanism in the aleurone layers. Compared with PEG + H_2_O_2_ treatment, the addition of DPI, an H_2_O_2_ synthesis specific inhibitor, decreased cellular H_2_O_2_ by 11%, while the addition of the H_2_O_2_ scavengers AsA and DMTU decreased cellular H_2_O_2_ by 11 and 13%, respectively (**Figure 2A**), implying that the production of cellular H_2_O_2_ induced by drought stress was scavenged or inhibited by AsA and DMTU and DPI, respectively. Following the addition of the HO-1 inducer hematin to the PEG treatment, cellular H_2_O_2_ declined by 9% compared with the PEG + H_2_O_2_ treatment, indicating that HO-1 could mimic the effects of AsA, DMUT, or DPI on scavenging or inhibition of cellular H_2_O_2_ (**Figure 2A**). However, in the PEG + H_2_O_2_ + ZnPPIX treatment containing the HO-1 inhibitor, ZnPPIX reversed the effect of HO-1. When hematin was added to PEG + H_2_O_2_ + AsA, PEG + H_2_O_2_ + DMTU, and PEG + H_2_O_2_ + DPI, the cellular H_2_O_2_ contents of the layers declined, while the HO-1 inhibitor ZnPPIX reversed the effect (**Figure 2A**).

To further determine whether endogenous H_2_O_2_ affects *HO-1* transcript expression of aleurone layers in response to drought stress, the cytosolic H_2_O_2_ levels were assessed. In this study, the aleurone layers treated for 24 h were labeled with the H_2_O_2_ fluorescence probe H2DCF-DA, and the fluorescence intensity of H_2_O_2_ was subsequently observed under LSCM (**Figures 2B–O**). The H_2_O_2_ fluorescence intensity of the aleurone cells treated with PEG alone was significantly higher than those treated with distilled water (**Figures 2B,C**), indicating that drought stress significantly promoted the H_2_O_2_ production in aleurone cells. Surprisingly, the H_2_O_2_ fluorescence intensity of the aleurone cells treated with PEG + H_2_O_2_ was 25% lower than those treated with PEG alone (**Figures 2C,D**), implying that the addition of exogenous H_2_O_2_ as a signaling molecule regulates the amount of endogenous H_2_O_2_ in the rice aleurone layer induced by drought stress. After the H_2_O_2_ scavengers AsA and DMTU and the H_2_O_2_ synthesis inhibitor DPI were added to the PEG+H_2_O_2_ treatment, the H_2_O_2_ fluorescence intensity decreased by 15, 13, and 12%, respectively (**Figures 2G,J,M**), indicating that AsA, DMTU, and DPI suppress the H_2_O_2_ production of aleurone cells under drought stress to some extent. Furthermore, after hematin was added to the PEG + H_2_O_2_, PEG + H_2_O_2_ + AsA, PEG + H_2_O_2_ + DMTU, and PEG + H_2_O_2_ + DPI treatments, the H_2_O_2_ fluorescence intensity of the cells weakened (**Figures 2E,H,K,N**), and ZnPPIX was able to reverse the effects of hematin (**Figures 2F,I,L,O**). The experimental results illustrated that the HO-1 inducer, H_2_O_2_ scavengers, and synthesis inhibitor effectively reduced the endogenous H_2_O_2_ levels of aleurone cells under drought stress (**Figure 2P**). Under drought conditions, exogenously applied H_2_O_2_ can support the induction of scavenging mechanisms which lead to reduced endogenous H_2_O_2_ levels.

### Exogenous H_2_O_2_ Assisted by HO-1 Enhanced the Amylase Activity in Aleurone Layers during Drought Stress

**FIGURE 3:**
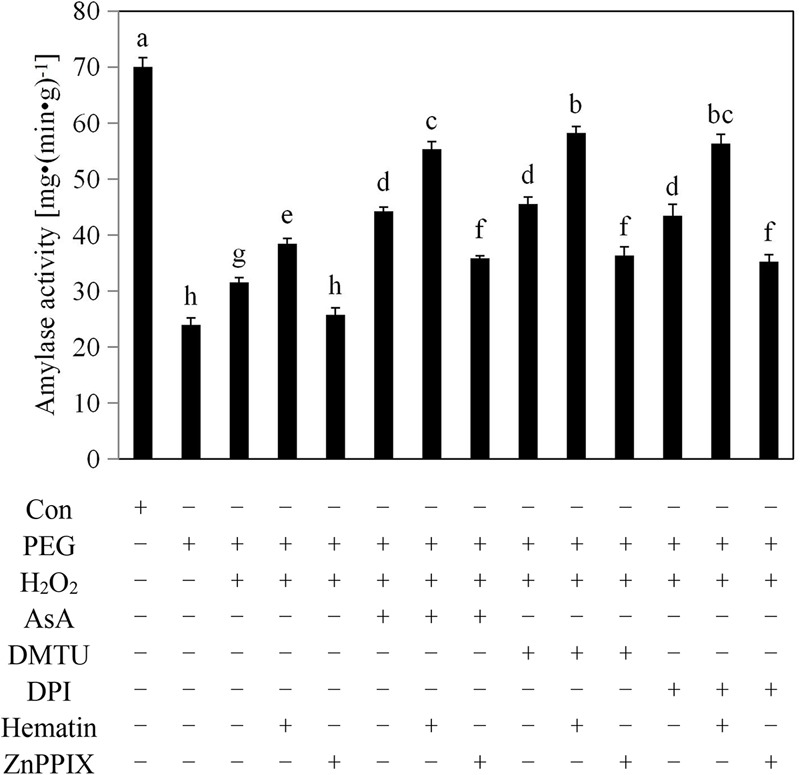
Exogenous H_2_O_2_, hematin, AsA, DMTU, and DPI raised the amylase activities of rice aleurone layers subjected to drought stress. Rice aleurone layers were incubated in a solution containing distilled water (Con) alone or 20% PEG, 1 mM H_2_O_2_, 5 mM AsA, 5 mM DMTU, 10 μM DPI, 1 μM hematin, and 10 μM ZnPPIX alone or a combination thereof for 24 h. Data are the means ± SD of three independent experiments with at least three replicates for each. Within each set of experiments, bars denoted by the different letters are significantly different to the Con treatment at *P* < 0.05 according to Duncan’s multiple test.

The aleurone layer secretes amylases into the starchy endosperm, which degrades the starch and provides nutrients for the embryo during the germination of cereal seeds. However, low levels of amylase are unable to degrade starch, resulting in the failure of the cereal seeds to germinate normally due to restricted embryo growth. As shown in **Figure 3**, treatment with PEG decreased the amylase activity of the aleurone layers by 64% compared with the distilled water treatment, suggesting that the amylase activity of the aleurone layers was significantly inhibited by drought stress. However, the activity of amylase in the PEG + H_2_O_2_-treated aleurone layers was higher than that during the PEG treatment, indicating that the application of an appropriate amount of H_2_O_2_ may help to induce amylase activity in rice aleurone layers under drought stress. The PEG + H_2_O_2_ + AsA, PEG + H_2_O_2_ + DMTU, and PEG + H_2_O_2_ + DPI treatments raised the amylase activities of the aleurone layers by 34, 27, and 41%, respectively, compared with the PEG + H_2_O_2_ treatment, indicating that scavenging or suppressing drought-induced H_2_O_2_ production improved the amylase activity of rice aleurone layers. This suggests that H_2_O_2_ plays a dual role in its effect on the amylase activity of rice aleurone layers; as a signaling molecule, H_2_O_2_ not only upregulated the amylase activity of the aleurone layers under drought stress but also, as an ROS, inhibited the amylase activity of layers under treatment with PEG alone. The PEG + H_2_O_2_ + hematin, PEG + H_2_O_2_ + AsA + hematin, PEG + H_2_O_2_ + DMTU + hematin, and PEG + H_2_O_2_ + DPI + hematin treatments increased the amylase activities of the aleurone layers compared with the treatments without hematin, and these effects were reversed by the HO-1 inhibitor ZnPPIX, implying that HO-1 assisted by exogenous H_2_O_2_ induced the amylase activity of rice aleurone layers in response to drought stress.

### HO-1 Cooperation with NO Delayed the Process of PCD in the Drought-Induced Aleurone Layers

**FIGURE 4:**
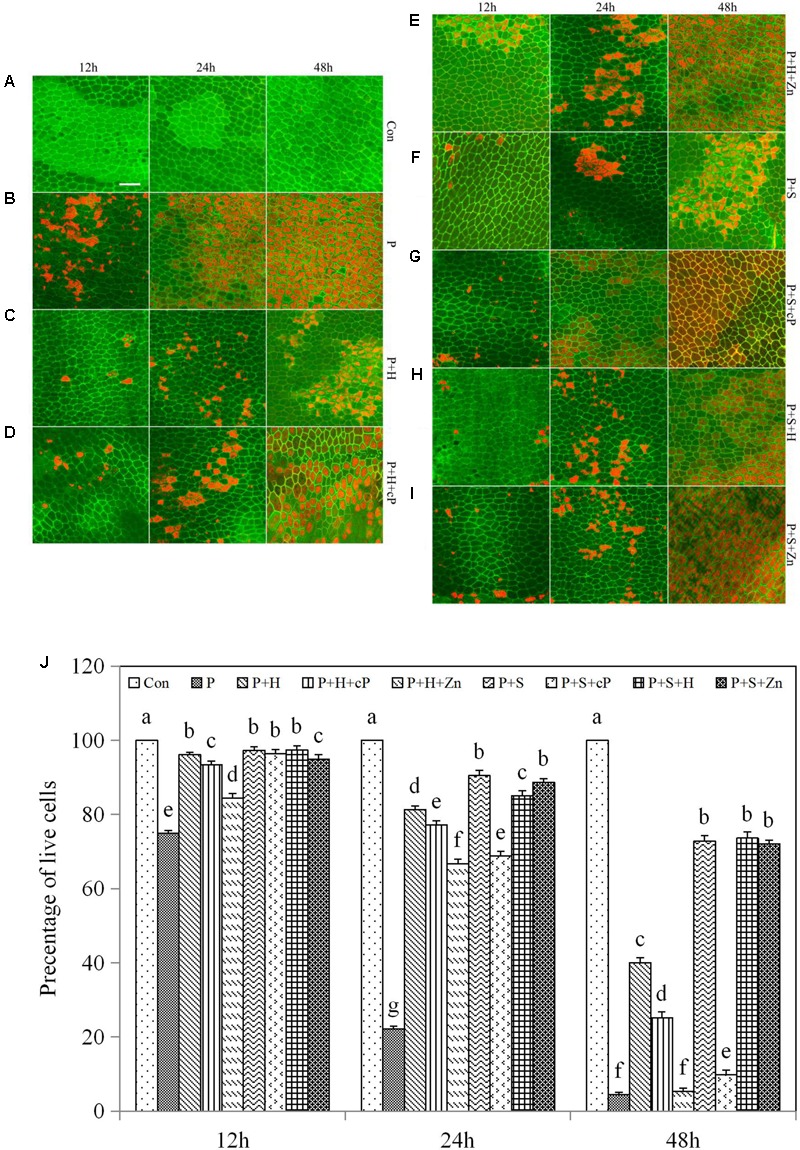
Hematin, SNP, ZnPPIX, and cPTIO promoted or delayed the PCD of rice aleurone layers under drought stress. Rice aleurone layers were incubated in a solution containing distilled water (Con) alone or 20% PEG (P), 1 μM hematin (H), 200 μM SNP (S), 10 μM ZnPPIX (Zn), and 200 μM cPTIO (cP) alone or in combination for 12, 24, and 48 h, respectively. After various treatments, the live and dead cells in the aleurone layers stained with FM-4-64 (orange or red, dead cells) and FDA (green, live cells) were observed by LSCM, and images were immediately captured **(A–I)**. In addition, cell survival rate was quantified **(J)** at 12, 24, and 48 h. Scale bar, 50 μM. Data are the means ± SD of three independent experiments with at least three replicates for each. Within each set of experiments, bars denoted by the different letters are significantly different to the Con treatment at *P* < 0.05 according to Duncan’s multiple test.

To determine the synergistic relationship between HO-1 and NO in drought-induced PCD of aleurone layers, we detected the cell viability of the layers using FDA/FM4-64 fluorescence dyes. Under an elongated culture period, the survival rate of aleurone cells remained at almost 100% in the distilled water treatment and showed a downward trend in the other treatments including PEG; cell death was greater in the latter than in the former after treatment for 24 and 48 h (**Figures 4A–J**). The viability rate of the aleurone cells was 74.88% in the PEG treatment at 12 h, and 22.15 and 4.43% at 24 and 48 h, respectively (**Figure 4B**), showing that cell death accelerated significantly in the layers under drought stress. However, the survival rate of aleurone cells treated with PEG + hematin was still 40.00% at 48 h (**Figure 4C**), while the PEG + hematin treatment containing ZnPPIX or cPTIO was about 66.67 or 77.17% at 24 h, respectively, and only 5.30 or 25.12% at 48 h, respectively (**Figures 4D,E**). The results demonstrated that hematin delays the occurrence of PCD in rice aleurone layers subjected to drought stress, and the effects are reversed by its inhibitor and NO scavengers.

Compared with treatment with PEG alone, the numbers of surviving cells in the rice aleurone layers treated with PEG + SNP improved by 22.40 and 68.31% at 12 and 24 h, respectively, and 68.42% at 48 h (**Figure 4F**). As observed with the HO-1 inducer hematin, the NO donor SNP effectively postponed the drought-induced PCD of rice aleurone layers. In comparison with treatment with PEG + SNP, the survival rate of aleurone cells in the PEG + SNP treatment containing hematin remained unchanged at 12 and 48 h and decreased slightly at 24 h (**Figures 4F,H**). In comparison to the PEG + hematin treatment, the cell survival rate in the PEG + SNP + hematin treatment increased by 1.30, 3.72, and 33.62% at 12, 24, and 48 h, respectively (**Figures 4C,H**). Furthermore, after the NO scavenger cPTIO was added to the PEG + SNP treatment, the survival rate of the aleurone cells decreased, particularly at 48 h, where the survival rate was only 9.85% (**Figure 4G**). However, after the addition of ZnPPIX, the survival rate was 88.67 and 72.10% at 24 and 48 h, respectively (**Figure 4I**), indicating that the NO donor SNP could reverse the drought-induced PCD of aleurone layers, but that it could not be reversed by the HO-1 synthase inhibitor ZnPPIX.

In summary, it is apparent that HO-1 and NO delay drought-induced PCD in the aleurone layers, and the effect of HO-1 on delaying PCD was reversed by the NO scavenger; however, the effect of NO on slowing PCD was not blocked by the HO-1 synthase inhibitor. The above results, combined with our previous results (Wu et al., 2016), confirm that HO-1 not only mediates NO but also is mediated by NO; therefore, HO-1 delays PCD of rice aleurone layers subjected to drought stress by cooperating with NO.

### NO Extended the Occurrence of PCD in the Drought-Induced Aleurone Layers by Mediating cGMP Pathway

**FIGURE 5:**
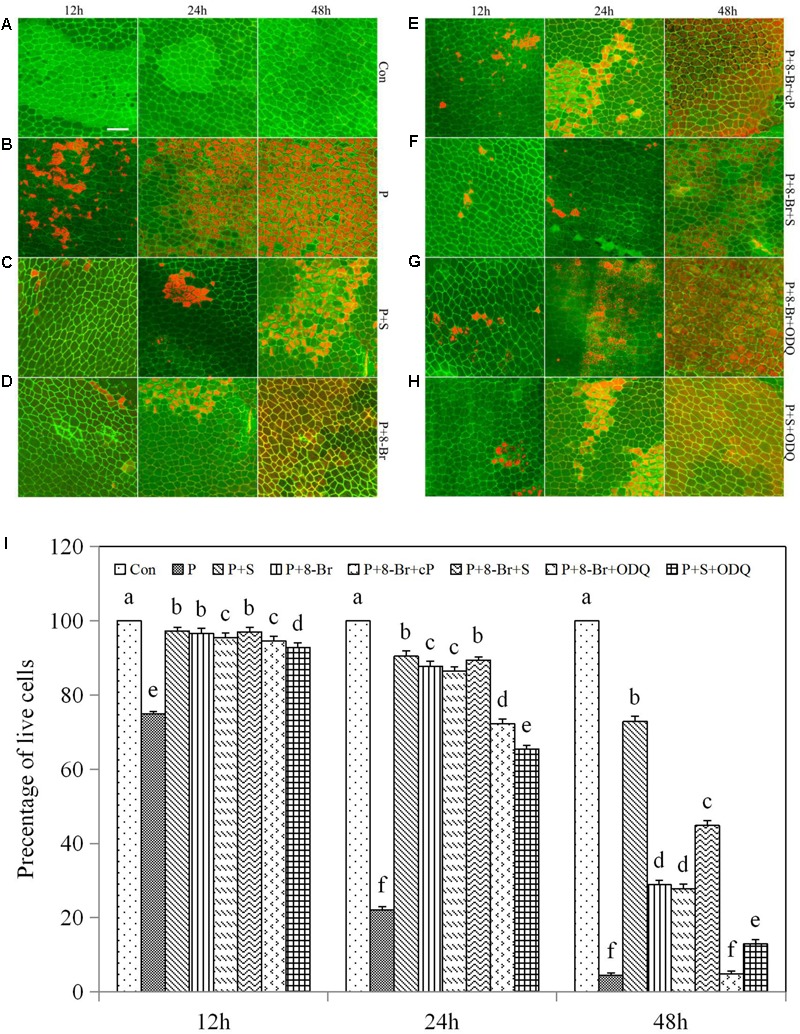
NO and cGMP regulated the PCD of rice aleurone layers subjected to drought stress. Rice aleurone layers were incubated in a solution containing distilled water (Con) alone or 20% PEG (P), 200 μM SNP (S), 200 μM cPTIO (cP), 10 μM 8-Br-cGMP (8-Br), and 10 μM ODQ alone or in combination for 12, 24, and 48 h, respectively. After various treatments, the live and dead cells in the aleurone layers stained with FM-4-64 (orange or red, dead cells) and FDA (green, live cells) were observed by LSCM, and images were immediately captured **(A–H)**. In addition, cell survival rate was quantified **(I)** at 12, 24, and 48 h. Scale bar, 50 μM. Data are the means ± SD of three independent experiments with at least three replicates for each. Within each set of experiments, bars denoted by the different letters are significantly different to the Con treatment at *P* < 0.05 according to Duncan’s multiple test.

cGMP is usually necessary for NO signal transduction (McDonald and Murad, 1995; Misra et al., 2010). To further explain the possible relationship between NO and the secondary messenger molecule cGMP in drought-induced PCD in rice aleurone layers, we applied the NO donor SNP and inhibitor cPTIO, the cGMP analog 8-Br-cGMP, and the synthesis inhibitor ODQ to PEG-treated aleurone layers (**Figures 5A–I**). The cell survival rate in the PEG treatment containing 8-Br-cGMP increased by 21.71, 66.59, and 24.44% in comparison with the PEG treatment alone at 12, 24, and 48 h, respectively (**Figures 5B,D**), suggesting that cGMP delays drought-induced PCD. When ODQ was added to the PEG + 8-Br-cGMP treatment, the survival rate of the aleurone cells was reduced 4.85% after 48 h of treatment (**Figure 5G**); after ODQ was replaced by cPTIO, the survival rate remained almost unchanged compared with the treatment of PEG+8-Br-cGMP (**Figure 5E**), indicating that the effect of cGMP on delaying drought-induced PCD of aleurone layers can be reversed by the guanylate cyclase (GC) synthesis inhibitor, but not the NO scavenger.

The cell survival rate was higher in the PEG + 8-Br-cGMP + SNP treatment than in the PEG + 8-Br-cGMP treatment and reached 97.00, 89.44, and 44.85% at 12, 24, and 48 h, respectively (**Figure 5F**), indicating a superposition effect between cGMP and NO. Therefore, in each time period, the cell survival rate was much lower in the PEG + SNP + ODQ treatment than in the PEG + SNP treatment (**Figures 5C,H**), indicating that the GC synthesis inhibitor ODQ can reverse the effect of NO in the attenuation of drought-induced PCD in rice aleurone layers. It is speculated that cGMP and NO delay the PCD of rice aleurone layers subjected to drought stress, and cGMP may act as a downstream component of NO.

### Exogenous H_2_O_2_ Suppressed the PCD of Aleurone Layers Exposed to Drought Stress in a cGMP-Dependent Manner

**FIGURE 6:**
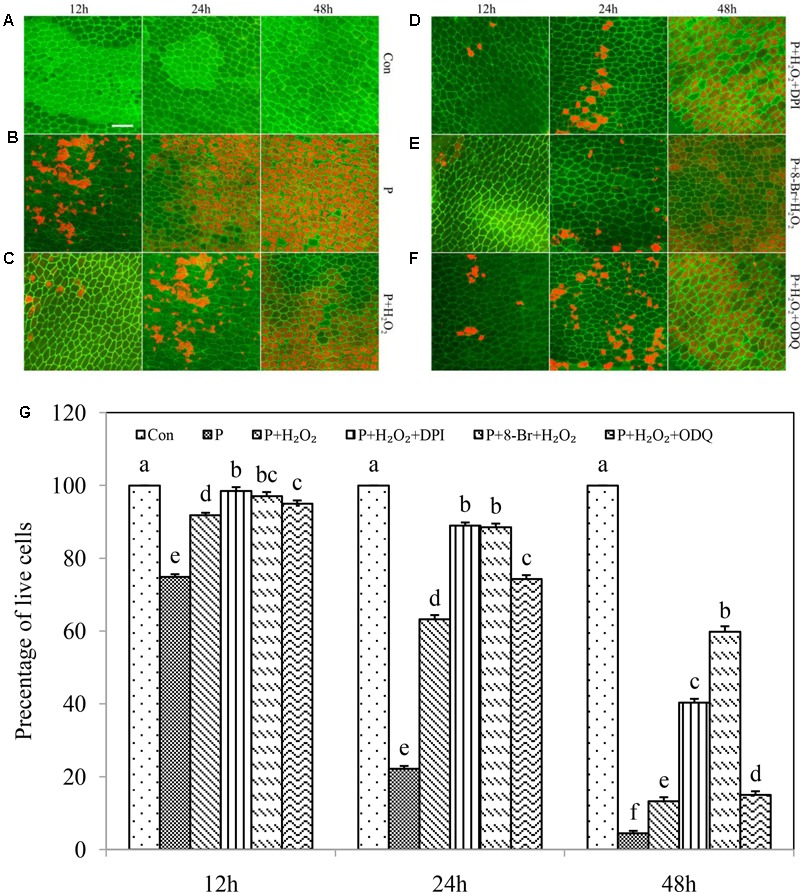
Exogenous H_2_O_2_ required cGMP for regulating PCD of rice aleurone layers subjected to drought stress. Rice aleurone layers were incubated in a solution containing distilled water (Con) alone or 20% PEG (P), 1 mM H_2_O_2_, 10 μM DPI, 10 μM 8-Br-cGMP (8-Br), and 10 μM ODQ alone or in combination for 12, 24, and 48 h, respectively. After various treatments, the live and dead cells in the aleurone layers stained with FM-4-64 (orange or red, dead cells) and FDA (green, live cells) were observed by LSCM, and images were immediately captured **(A–F)**. In addition, cell survival rate was quantified **(G)** at 12, 24, and 48 h. Scale bar, 50 μM. Data are the means ± SD of three independent experiments with at least three replicates for each. Within each set of experiments, bars denoted by the different letters are significantly different to the Con treatment at *P* < 0.05 according to Duncan’s multiple test.

Exogenous H_2_O_2_ may be involved in activating 3-O-C10-HL-induced cGMP synthesis in the adventitious root formation of mung bean (Bai et al., 2012). Given this, we wanted to investigate whether exogenous H_2_O_2_ regulates the PCD of aleurone layers subjected to drought stress via the cGMP pathway (**Figures 6A–G**). The survival rate of aleurone cells in PEG alone was 74.88, 22.15, and 4.43% at 12, 24, and 48 h (**Figure 6B**) and 91.84, 63.21, and 31.07% in the PEG + H_2_O_2_ treatment, respectively (**Figure 6C**). In addition, the survival rate of aleurone cells in the PEG + H_2_O_2_ treatment containing the H_2_O_2_ synthesis inhibitor DPI was much higher than in the PEG + H_2_O_2_ treatment: 98.50% at 12 h and 87.99% at 24 h (**Figure 6D**). However, the survival rate after PEG + H_2_O_2_ + DPI treatment was lower than that after PEG + H_2_O_2_ treatment at only 18.26% at 48 h (**Figure 6D**). It is possible that as time progressed, the amount of DPI was insufficient to inhibit the synthesis of endogenous H_2_O_2_, leading to H_2_O_2_ bursts and further cell damage. Furthermore, the survival rate of aleurone layers in the treatment of PEG + H_2_O_2_ + 8-Br-cGMP increased by 5.24, 25.29, and 28.75% at 12, 24, and 48 h, respectively, compared with that of PEG + H_2_O_2_ (**Figure 6E**). after the GC synthesis inhibitor ODQ was added, the PEG + H_2_O_2_ + ODQ treatment reversed the effect of PEG + H_2_O_2_ + 8-Br-cGMP (**Figure 6F**), further indicating that the addition of exogenous H_2_O_2_ as a signaling molecule participates in regulating the PCD of drought-induced rice aleurone layers by mediating the cGMP pathway.

## Discussion

H_2_O_2_ was previously thought to be toxic to cells, but increasing evidence now suggests that H_2_O_2_ is a key signaling molecule involved in various physiological activities in plants, particularly protecting plant cells against abiotic stresses (Gechev et al., 2002; Azevedo-Neto et al., 2005; Guler and Pehlivan, 2016). Interestingly, due to the application of exogenous H_2_O_2_, the tolerance to abiotic stress including drought, salinity, and heavy metal salt, all of which caused elevated H_2_O_2_ levels, was enhanced in plants (Chao et al., 2009; Gondim et al., 2012; Hossain and Fujita, 2013). In most cases, the involvement of exogeous H_2_O_2_ could increase the activities of the antioxidant enzymes CAT, SOD, APX, GPOX, DHAR, GR, and the levels of the antioxidants AsA and GSH, resulting in decreased levels of endogenous H_2_O_2_ in plants under drought stress (Jing et al., 2009; Liu Z.J. et al., 2010; Hossain and Fujita, 2013). Furthermore, the investigations of Lecube et al. (2014) and Wu et al. (2016) showed the beneficial role of HO-1 in soybean and rice subjected to drought stress. Importantly, the synergistic effects of exogenous H_2_O_2_ and HO-1 were observed in plants subjected to oxidative stress (Yannarelli et al., 2006; Chen et al., 2009). Low concentrations of H_2_O_2_ increased *HO-1* transcript expression and HO activity in wheat seedling leaves in response to oxidative stress (Chen et al., 2009), and H_2_O_2_ mediated by HO-1 induced the formation of lateral roots in apocynin-treated rice (Chen et al., 2013). In this study, exogenous H_2_O_2_ up-regulated *HO-1* transcript levels (**Figure 1**) and the up-regulation of *HO-1* expression contributed to lowering cellular H_2_O_2_ levels (**Figure 2**) and promoting amylase activities (**Figure 3**). As expected, the beneficial effect of exogenous H_2_O_2_ in rice aleurone layers is supported by some studies that showed, for example, that exogenous application of H_2_O_2_ enhanced the activities of the antioxidant enzymes SOD, CAT, and APX, and also reduced the content of MDA in wheat and maize subjected to salt stress (Li et al., 2011; Gondim et al., 2012), and that endogenous H_2_O_2_ levels were not enhanced by the addition of a low concentration H_2_O_2_ in seedlings for 24 or 48 h prior to drought stress or salt stress (Fedina et al., 2009; Hossain and Fujita, 2013). Subsequent experiments revealed that the HO-1 inducer hematin can mimic the effects of the antioxidants AsA and DMTU on down-regulating endogenous H_2_O_2_ levels (**Figure 2**), indicating that HO-1 may act as a potent antioxidative enzyme in the protection of cells from oxidative stress (Wu et al., 2011, 2014). Our experiments also revealed that DPI, an NADPH synthase inhibitor, significantly inhibited H_2_O_2_ levels (**Figure 2**). In addition, as observed with hematin, the H_2_O_2_ scavengers AsA and DMTU and the synthesis inhibitor DPI could up-regulate *HO-1* transcript expression. This finding is in agreement with previously reported results showing that the antioxidants AsA, dithiothreitol (DTT), and hydroxytoluene (BHT) mimic the effect of the HO-1 inducer hematin by up-regulating *HO-1* transcript levels and delaying PCD in wheat aleurone layers (Wu et al., 2011). More importantly, the HO-1 inducer hematin enhanced the effects of AsA, DMTU, and DPI toward exogenous H_2_O_2_-upregulated *HO-1* transcript levels and amylase activity, and down-regulated endogenous H_2_O_2_ production in rice aleurone layers in response to drought stress. In contrast, the HO-1 inhibitor ZnPPIX blocked these effects.

In addition, our experiment further indicated that exogenous H_2_O_2_ delayed the PCD of rice aleurone layers in response to drought stress, and this delaying effect was enhanced by the endogenous H_2_O_2_ synthesis inhibitor DPI, indicating that endogenous H_2_O_2_ produced by drought stress severely damages cells, resulting in PCD (**Figure 6**). This beneficial H_2_O_2_ effect was also observed by Neill et al. (2003), who suggested that the exogenous addition of H_2_O_2_ reduces cellular damage caused by low temperatures, thereby increasing the survival rate. Therefore, it is crucial that the balance between the production and scavenging of H_2_O_2_ is precisely regulated by cell antioxidant machinery, which determines whether H_2_O_2_ acts as a signaling molecule delaying PCD, or as a toxic oxidative molecule promoting PCD.

As an endogenous secondary messenger, cyclic guanosine monophosphate (cGMP) is formed through guanylate cyclase (GC) catalysis and guanosine monophosphate (GTP) hydrolysis. H_2_O_2_ and NO contribute toward the increase in GC activity in plants (Dubovskaya et al., 2011; Mulaudzi et al., 2011). H_2_O_2_ plus the cGMP analog 8-Br-cGMP was found to delay PCD of the layers in response to drought stress, and the effect of cGMP-delayed PCD was reversed by the GC synthesis inhibitor ODQ (**Figure 6**). It appears that cGMP acts downstream of H_2_O_2_ in regulating the PCD of rice aleurone layers during drought stress. This finding is similar to previous studies in which H_2_O_2_ and NO increased the concentration of cyclic nucleotides, which can improve the activity of GC in plants (Dubovskaya et al., 2011; Mulaudzi et al., 2011). Moreover, it has been demonstrated that cGMP regulates the expression of the α-amylase gene in tobacco aleurone layers (Durner et al., 1998), and the effect of LY83583 on α-amylase was reversed by cGMP analogs (Penson et al., 1996). Furthermore, PCD occurred later than the mRNA expression of α-amylase in barley aleurone layers (Penson et al., 1996). In combination with the inhibition of cGMP in inducing PCD of rice aleurone layers during drought stress, this indicates that cGMP regulates the PCD process of isolated aleurone cells in the upstream of amylase.

NO exerts a protective effect on the growth of wheat seedlings under drought stress (Tian and Lei, 2006) and also alleviates low-temperature stress (Zhao et al., 2009; Tan et al., 2013; Liu et al., 2016). In addition, NO was found to delay the PCD of aleurone layers in barley (Beligni et al., 2002), and the transcript expression of *HO-1* in plants is also regulated by NO (Noriega et al., 2007; Santa-Cruz et al., 2010). In this experiment, HO-1 delayed the PCD in the drought-induced rice aleurone layers, and this effect was reversed by its synthesis inhibitor ZnPPIX and the NO scavenger cPTIO. However, the effect of NO on delaying PCD was reversed only by its scavenger cPTIO and not by ZnPPIX (**Figure 4**). It appears that NO acts downstream of HO-1 and is involved in delaying the PCD of rice aleurone layers during drought stress. However, our previous results also confirmed that the HO-1 inducer hematin induced the production of endogenous NO in rice aleurone layers subjected to drought stress, and correspondingly, the HO-1 synthesis inhibitor ZnPPIX reduced endogenous NO (Wu et al., 2016). Therefore, we suggest that HO-1 delays the PCD of aleurone layers subjected to drought stress by interacting with NO. Moreover, NO ordinarily requires cGMP, as a downstream molecule, to be involved in the signaling pathway (Pagnussat et al., 2003; Desikan et al., 2004; Cao et al., 2007). When plants are subjected to stress, NO-induced cGMP was found to increase (Garcia-Mata et al., 2003). Our experimental evidence also showed that the effect of NO-delayed PCD in rice aleurone layers was reversed by its scavenger cPTIO and the GC synthesis inhibitor ODQ during drought stress, and the effect of cGMP-delayed PCD was reversed by the GC synthesis inhibitor ODQ, not by the NO scavenger cPTIO (**Figure 5**). The results demonstrated that cGMP acts downstream of NO, and combining the relationship between HO-1 and NO in regulating the PCD of rice aleurone layers subjected to drought stress, we speculated that HO-1 and NO regulate the PCD of rice aleurone layers via a cGMP-dependent signaling pathway.

**FIGURE 7:**
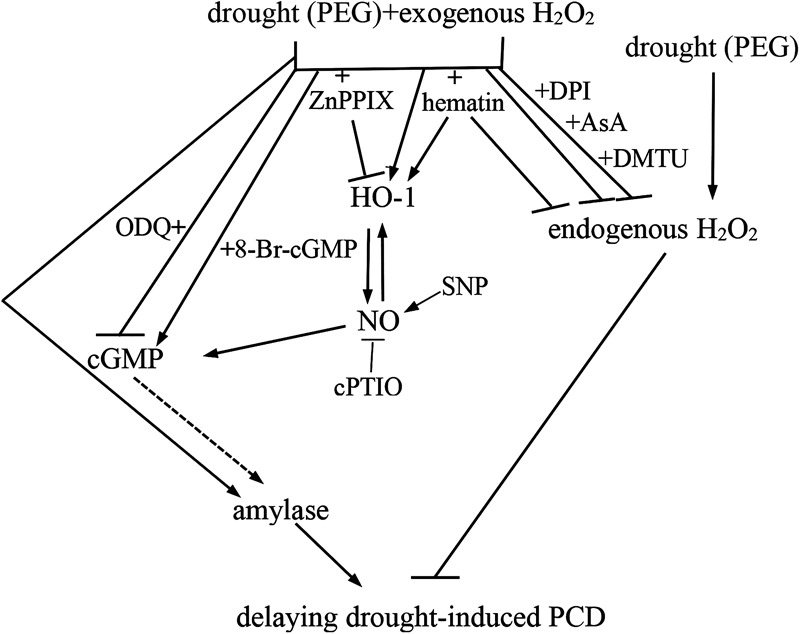
Schematic representation of the signaling pathway involving H_2_O_2_, HO-1, NO, cGMP, and amylase during drought-induced PCD of isolated aleurone layers in rice. Dashed line denotes the possible relationship between cGMP and amylase. T bars denote inhibition.

To sum up, we confirmed that exogenous H_2_O_2_ significantly delayed the PCD of rice aleurone layers under drought stress, and HO-1 played an important role in this process. We also indicated that the reduced levels of intracellular H_2_O_2_ was beneficial to the alleviation of drought stress-induced PCD. More importantly, exogenous H_2_O_2_ was able to up-regulate HO-1, which in turn inhibited the production of endogenous H_2_O_2_, finally, resulting in delaying the PCD of rice aleurone layers subjected to drought stress. Similarly, the up-regulation of *HO-1* gene transcript was observed in exogenous H_2_O_2_-treated wheat plant in oxidative stress (Chen et al., 2009). Additionally, previous reports have revealed that up-regulating HO-1 suppressed the endogenous H_2_O_2_ production in GA-treated wheat aleurone layers by increasing APX and CAT activities (Wu et al., 2011), and delayed the GA-induced PCD of rice aleurone layers subjected to drought stress (Wu et al., 2016). In this study, the up-regulation of HO-1 promoted the increase of amylase activity in rice aleurone layers subjected to drought stress. It is noteworthy that, the up-regulation of HO-1 triggered NO, and then NO induced cGMP-mediated *α-Amy2/54* gene expression and amylase activity in GA-treated wheat aleurone layers (Wu et al., 2013). Our previous evidence also revealed that HO-1 delayed the GA-induced PCD by cooperating with NO in rice aleurone layers in response to drought stress (Wu et al., 2016). Based on the effects of both NO and cGMP on delaying drought-induced PCD, we deducted that HO-1 triggered NO and thereafter NO activated cGMP. Furthermore, H_2_O_2_ regulated the PCD process of rice aleurone layers subjected to drought stress in a cGMP-dependent manner. Therefore, all above results suggested that exogenous H_2_O_2_-triggered up-regulation of *HO-1* gene expression play a vital role in delaying drought stress-induced PCD by cooperating with NO mediated by cGMP via amylase (**Figure 7**).

## Author Contributions

HC designated the experiment. HC and GW wrote the paper. GW and YX contributed in initial drafting of the manuscript, developing some of the figures, and have contributed equally. XD, HZ, and TL helped in drafting the manuscript.

## Conflict of Interest Statement

The authors declare that the research was conducted in the absence of any commercial or financial relationships that could be construed as a potential conflict of interest.
